# Cell-Free DNA (cfDNA) Regulates Metabolic Remodeling, Sustaining Proliferation, Quiescence, and Migration in MDA-MB-231, a Triple-Negative Breast Carcinoma (TNBC) Cell Line

**DOI:** 10.3390/metabo15040227

**Published:** 2025-03-27

**Authors:** Isabel Lemos, Catarina Freitas-Dias, Ana Hipólito, José Ramalho, Fabrizio Carteni, Luís G. Gonçalves, Stefano Mazzoleni, Jacinta Serpa

**Affiliations:** 1 iNOVA4Health, NOVA Medical School, Faculdade de Ciências Médicas, Universidade NOVA de Lisboa, Campo dos Mártires da Pátria, 130, 1169-056 Lisbon, Portugal; a2022507@nms.unl.pt (I.L.); catarina.freitasdias@nms.unl.pt (C.F.-D.); ana.hipolito@nms.unl.pt (A.H.); jose.ramalho@nms.unl.pt (J.R.); 2 Instituto Português de Oncologia de Lisboa Francisco Gentil (IPOLFG), Rua Prof Lima Basto, 1099-023 Lisbon, Portugal; 3 Lab Applied Ecology and System Dynamics, Dipartimento di Agraria, Università di Napoli “Federico II”, Portici, 80055 Naples, Italy; fabrizio.carteni@unina.it (F.C.); stefano.mazzoleni@unina.it (S.M.); 4 Instituto de Tecnologia Química e Biológica António Xavier (ITQB NOVA), Avenida da República (EAN), 2780-157 Oeiras, Portugal; lgafeira@itqb.unl.pt

**Keywords:** cell-free DNA (cfDNA), metabolic remodeling, cell proliferation, quiescence, migration, toll-like receptor 9 (TLR9), cancer cell selection

## Abstract

**Background:** The clinical relevance of circulating cell-free DNA (cfDNA) in oncology has gained significant attention, with its potential as a biomarker for cancer diagnosis and monitoring. However, its precise role in cancer biology and progression remains unclear. cfDNA in cancer patients’ blood has been shown to activate signaling pathways, such as those mediated by toll-like receptors (TLRs), suggesting its involvement in cancer cell adaptation to the tumor microenvironment. **Methods:** This impact of cfDNA released from MDA-MB-231, a triple-negative breast cancer (TNBC) cell line was assessed, focusing on glucose availability and culture duration. The impact of cfDNA on the proliferation of MDA-MB-231 cells was investigated using proliferation curves, while cellular migration was evaluated through wound healing assays. The metabolic alterations induced by distinct cfDNA variants in MDA-MB-231 cells were investigated through nuclear magnetic resonance (NMR) spectroscopy, and their effect on cisplatin resistance was evaluated using flow cytometry. Furthermore, the expression levels of DNA-sensitive Toll-like receptor 9 (TLR9) were quantified via immunofluorescence, alongside its colocalization with lysosome-associated membrane protein 1 (LAMP1). **Results:** This study indicates that cfDNA facilitates metabolic adaptation, particularly under metabolic stress, by modulating glucose and glutamine consumption, key pathways in tumor cell metabolism. Exposure to cfDNA induced distinct metabolic shifts, favoring energy production through oxidative phosphorylation. The anti-cancer activity of cfDNA isolated from conditioned media of cells cultured under stressful conditions is influenced by the culture duration, emphasizing the importance of adaptation and se-lection in releasing cfDNA that can drive pro-tumoral processes. Additionally, cfDNA exposure influenced cell proliferation, quiescence, and migration, processes linked to metastasis and treatment resistance. These findings underscore cfDNA as a key mediator of metabolic reprogramming and adaptive responses in cancer cells, contributing to tumor progression and therapy resistance. Furthermore, the activation of TLR9 signaling suggests a mechanistic basis for cfDNA-induced phenotypic changes. **Conclusions**: Overall, cfDNA serves as a crucial signaling molecule in the tumor microenvironment, orchestrating adaptive processes that enhance cancer cell survival and progression.

## 1. Introduction

Cell-free DNA (cfDNA) is defined as extracellular DNA fragments that are released into body fluids, such as the blood, urine, and saliva [1]. cfDNA can be detected in fluids of healthy people at varied but low concentrations [2]. Elevated cfDNA concentrations could be associated both with specific pathological and non-pathological contexts, such as tissue cancer, trauma, autoimmune diseases, and infections [3,4,5,6], or even intense physical exercise [7]. In cancer settings, many studies have reported elevated concentrations of cfDNA in blood serum when compared to healthy controls [8,9,10]. Thus, cfDNA derived from cancer patients’ peripheral blood has been explored as a biomarker, aiming to improve disease management, especially given the existence of tumor-specific somatic mutation burden [11,12,13,14]. The use of cfDNA as a biomarker could, therefore, allow early screening and reliable prognosis and follow-up of cancer patients [15], with low overall costs and in a minimally invasive way.

cfDNA has already been pointed out as a key driver of cancer metastasis [16,17]; however, the precise methods by which cfDNA regulates cancer cell characteristics required for disease development, such as cancer metabolic remodeling, remain unknown. Breast cancer (BC) is a highly heterogeneous disease comprising different molecular subtypes [18]. These subtypes are often classified into four main groups based on the expression of hormone receptors, namely estrogen (ER) and progesterone receptor (PR), together with the epidermal growth factor receptor 2 (HER2) [19,20]. Similar to other malignancies, patients with BC were reported to have high levels of cfDNA [21,22]; however, cfDNA levels in triple-negative breast cancer (TNBC) patients significantly stand out among the remaining BC subtypes [23]. The TNBC subtype is distinguished by the absence of expression of any of the receptors and accounts for 10 to 20% of all BC [18,24]. TNBC is a heterogeneous group, presenting high aggressiveness and recurrence rates, as well as poor prognosis [24,25]. Despite its limited benefit, chemotherapy remains the accepted standard treatment for TNBC [26] since these tumors are known to be intrinsically chemo-sensitive. Yet, it is also prone to fast relapse and acquired resistance [24,27].

In this study, we investigated whether cfDNA produced by MDA-MB-231 cells, a TNBC cell line, contributes to a metabolic remodeling that promotes chemoresistance. In line with this, we investigated how the cfDNA released in the presence and absence of glucose at different cell culture timepoints would affect the phenotypic traits and metabolic profiles of MDA-MB-231 cells.

## 2. Materials and Methods

### 2.1. Cell Culture Conditions

Breast carcinoma cell line MDA-MB-231 (ATCC^®^ HTB-26™) was obtained from the American Type Culture Collection (ATCC). Cells were cultured in Dulbecco’s Modified Eagles’ Medium (DMEM; 41965-039, Gibco, Life Technologies, Waltham, MA, USA) supplemented with 10% fetal bovine serum (FBS; S0615, Merck, Rahway, NJ, USA), 1% Antibiotic-Antimycotic (AA; P06-07300, PAN Biotech, Aidenbach, Germany), and 50 µg/mL Gentamicin (15750-060, Gibco, Life Technologies, MA, USA). Cells were maintained in a humidified environment of 37 °C with 5% CO_2_ and maintained to 75–100% optical confluence. For detachment, 0.05% trypsin–ethylenediaminetetraacetic acid (EDTA) 1× (25300-054, Invitrogen, Waltham, MA, USA) was used.

### 2.2. Cell-Free DNA Isolation

MDA-MB-231 cells were cultured in T-75 flasks until ~80% of optical confluence was reached. DNA was isolated from the cell culture conditioned media with the following conditions: absence or presence of glucose (5 mM) during 6 or 48 h of cell culture. The QIAamp^®^ DNA Blood Mini Kit (51106, Qiagen, Hilden, Germany) was used to perform the isolation, according to the manufacturer’s indications. Four distinct cfDNA types were obtained based on the defined culture conditions: Early-cfDNA-Gluc (cells cultured with glucose for 6 h), Early-cfDNA-NoGluc (cells cultured without glucose for 6 h), Late-cfDNA-Gluc (cells cultured with glucose for 48 h), and Late-cfDNA-NoGluc (cells cultured without glucose for 48 h). The cfDNA used throughout this study was aliquoted after extraction and kept at −20 °C until use.

### 2.3. Cell Proliferation

MDA-MB-231 cells were seeded in 96-well plates (1 × 10^4^ cells/well) in complete DMEM and synchronized under starvation (FBS-free media) overnight. Cells were then exposed to the experimental conditions: 10 ng/mL of each different variants of cfDNA in the presence (5 mM) or absence of glucose. After 6 or 48 h of exposure, cells both adherent and in suspension were collected. The samples were then centrifuged at 155× *g* for 5 min. Cells were counted in a Neubauer enhanced counting chamber using Trypan Blue Staining (15250-061, Gibco), and the total number of cells was considered for the proliferation curves. Control cells were not exposed to cfDNA.

### 2.4. Wound Healing Assay

MDA-MB-231 cells were plated in 12-well plates (2.5 × 10^5^ cells/well) with complete DMEM and cultured until 70–80% optical confluence. Cells were starved (FBS-free media) overnight for synchronization and, after this, exposed to Mitomycin-C (5 µg/mL, M4287, Sigma, MA, USA) for 3 h. A scratch (wound) in the monolayer was induced with a 200 µL pipette tip. Cells were washed in 1× PBS and subjected to Late-cfDNA-Gluc and Late-cfDNA-NoGluc. Images of wound closure were captured at 0, 2, 6, 8, 10, 24, 28, 32, and 48 h in phase-contrast microscopy under an Olympus IX53 Inverted Microscope (State College, PA, USA), using Olympus cellSens v.1.17 software. Images were analyzed, and the wound closure was quantified using ImageJ v.1.53 software (https://imagej.net/, downloaded on 1 October 2023).

### 2.5. Nuclear Magnetic Resonance (NMR) Spectroscopy

MDA-MB-231 cells were cultured in T-75 Flasks for 6 and 48 h in glucose-free DMEM (P04-01549, Pan-Biotech, Aidenbach, Germany), supplemented with 1% AA and either with or without 5 mM D-glucose. The culture medium was collected and stored at −80 °C. MDA-MB-231 cells were plated in 24-well plates (1 × 10^5^ cells/well). Cells were starved (FBS-free media) overnight, then exposed to 5 mM D-glucose with Early-cfDNA-Gluc, Early-cfDNA-NoGluc, Late-cfDNA-Gluc, and Late-cfDNA-NoGluc for 48 h. Supernatants were stored at −80 °C after centrifugation at 150× *g* for 2 min, and pellets were discarded.

For NMR analysis, 540 µL of supernatant (conditioned media) sample, 30 µL of 0.4% (*v*/*v*) sodium azide in deuterated water (D_2_O), and 30 µL of a solution of 2.2 mM 3-(trimethylsilyl)propionic-2,2,3,3-d4 acid (TSP) were mixed, and TSP was used as an internal ^1^H-NMR quantification and chemical shift reference. ^1^H-NMR spectra were acquired at 25 °C using a Bruker Ultrashield^TM^ Avance 500 Plus spectrometer with a TCI-Z probe. The ^1^H-NMR was performed using a noesypr1d pulse program (128 scans, 3 s relaxation delay, 10 milliseconds mixing time, and 65 k points of free induction decay (FID). Spectra were acquired with TopSpin 4.1 software (Bruker), and assignments were determined utilizing spectral databases such as Human Metabolome (HMDB) and Chenomx NMR Suite 8.11.

### 2.6. Cell Death Analysis by Flow Cytometry

MDA-MB-231 cells were plated in 24-well plates (1 × 10^5^ cells/well) in complete DMEM and exposed to 10 ng/mL of Early-cfDNA-Gluc, Early-cfDNA-NoGluc, Late-cfDNA-Gluc, and Late-cfDNA-NoGluc for 24 h. Cells were maintained in normoxia or in hypoxia-mimicking conditions (0.1 mM CoCl_2_; (7646-79-9, Sigma-Aldrich, St. Louis, MA, USA), with and without 0.402 mM L-cysteine (102839, Merck). The experiment was performed with and without 25 µg/mL cisplatin for an extra 24 h. The culture media and the adherent cells were collected and centrifuged at 150× *g* for 3 min. The samples were incubated with 0.5 µL of Annexin V-fluorescein (FITC) (640906, BioLegend, San Diego, CA, USA) in 100 µL of annexin V binding buffer for 15 min at room temperature (RT) in the dark. Then, cells were washed with 200 µL 1× PBS containing 0.1% (*v*/*w*) BSA and centrifuged for 2 min at 150× *g*. Cells were resuspended in annexin V binding buffer 1× with 1.25 µL of propidium iodide (PI, 50 µg/mL; P4170, Sigma-Aldrich, St. Louis, MA, USA). Data were acquired in a BD Accuri^TM^ C6 flow cytometer from Becton Dickinson and analyzed using the BD Accuri Software v1.0.34.1.

### 2.7. Reactive Oxygen Species (ROS) Quantification by Flow Cytometry

MDA-MB-231 cells were plated in 24-well plates (5 × 10^4^ cells/well) in complete DMEM and stimulated with 10 ng/mL of Early-cfDNA-Gluc, Early-cfDNA-NoGluc, Late-cfDNA-Gluc, and Late-cfDNA-NoGluc for 24 h. Cells were maintained in normoxia or in hypoxia-mimicking conditions (0.1 mM CoCl_2_), with and without 0.402 mM L-cysteine. The experiment was performed with and without 25 µg/mL cisplatin for an extra 24 h. Cells were detached, collected, and incubated with a 10 μM DCF-DA probe (D6883, Sigma Aldrich, St. Louis, MA, USA) at 37 °C for 30 min. Data were acquired in a BD Accuri^TM^ C6 flow cytometer from Becton Dickinson and analyzed using the BD Accuri Software v1.0.34.1.

### 2.8. Lipid Peroxide Quantification by Flow Cytometry

MDA-MB-231 cells were plated in 24-well plates (5 × 10^4^ cells/well) in complete DMEM and stimulated with 10 ng/mL of Early-cfDNA-Gluc, Early-cfDNA-NoGluc, Late-cfDNA-Gluc, and Late-cfDNA-NoGluc for 24 h. Cells were maintained in normoxia or in hypoxia-mimicking conditions (0.1 mM CoCl_2_), with and without 0.402 mM L-cysteine. The experiment was performed with and without 25 µg/mL cisplatin for an extra 24 h. The culture media and the adherent cells were collected and incubated with 2 μM C11-Bodipy 581/591 (D3861, Invitrogen, Waltham, MA, USA) for 30 min at 37 °C in the dark. After washing with 1× PBS-2% FBS, pellets were resuspended in 1× PBS-2% FBS. Data were acquired in a BD Accuri^TM^ C6 flow cytometer from Becton Dickinson and analyzed using the BD Accuri Software v1.0.34.1.

### 2.9. Immunofluorescence

MDA-MB-231 cells were plated on 24-well plates (1 × 10^5^ cells/well) on glass coverslips coated with 0.2% gelatin from porcine skin (G-1890, Sigma-Aldrich, St. Louis, MA, USA). Cells were then cultured in complete DMEM and stimulated with 10 ng/mL of Early-cfDNA-Gluc, Early-cfDNA-NoGluc, Late-cfDNA-Gluc, and Late-cfDNA-NoGluc for 24 h. Cells were maintained in normoxia or in hypoxia-mimicking conditions (0.1 mM CoCl_2_), with and without 0.402 mM L-cysteine (102839, Merck, Rahway, NJ, USA). Cell fixation was performed with 4% paraformaldehyde at RT for 15 min. After, cells were incubated with 50 mM ammonium chloride (NH_4_Cl) for 10 min at 4 °C and permeabilized with saponin 0.1% in PBS 1×—0.5% BSA (*w*/*v*) for 15 min at RT. Cells were incubated with rabbit anti-human xCT (1:500, ab37185, Abcam, Cambridge, UK) in PBS 1× with 0.1% saponin—0.5% BSA (*w*/*v*) overnight at 4 °C. Then cells were rinsed twice with PBS 1× with 0.1% saponin—0.5% BSA (*w*/*v*), followed by incubation with Alexa Fluor 488 anti-rabbit (1:1000; A-11034, Invitrogen, Waltham, MA, USA) in PBS 1× with 0.1% saponin—0.5% BSA (*w*/*v*) for 2 h at RT.

We also detected the NRF2 protein by immunofluorescence. For this assay, MDA-MB-231 cells were stimulated with 10 ng/mL of Early-cfDNA-Gluc, Early-cfDNA-NoGluc, Late-cfDNA-Gluc, and Late-cfDNA-NoGluc for either 6 h or 48 h. Fixed cells were incubated with rabbit anti-human NRF2 (1:100; ab31163, Abcam, Cambridge, UK) in PBS 1× with 0.1% saponin—0.5% BSA (*w*/*v*) overnight at 4 °C. After being rinsed twice with PBS 1× with 0.1% saponin—0.5% BSA (*w*/*v*), cells were incubated with the secondary antibody Alexa Fluor 488 anti-rabbit (1:1000; A-11034, Invitrogen, Waltham, MA, USA) for 2 h at RT.

For TLR9 and LAMP1 detection, fixed cells were first incubated with mouse anti-TLR9 (1:200; MA5-38645, Invitrogen, Waltham, MA, USA) in PBS 1× with 0.1% saponin—0.5% BSA (w/v) overnight at 4 °C, and after being rinsed twice with PBS 1× with 0.1% saponin—0.5% BSA (*w*/*v*), incubation with the secondary antibody Alexa Fluor 488 anti-mouse (1:1000; 115-545-003, Invitrogen, Waltham, MA, USA) was performed. After this, cells were incubated with rabbit anti-human LAMP1 (1:200; MA5-29385, Invitrogen, Waltham, MA, USA) in PBS 1× with 0.1% saponin—0.5% BSA (*w*/*v*) for 3 h at RT. Slides were rinsed twice with PBS 1× with 0.1% saponin—0.5% BSA (*w*/*v*), and incubation with the secondary antibody Alexa Fluor 594 anti-rabbit (1:1000; A-11037, Invitrogen, Waltham, MA, USA) was performed.

Slides were mounted in VECTASHIELD media containing 4′-6-diamidino-2-phenylindole (DAPI) (Vector Labs, Newark, CA, USA). Images were captured and processed using a Zeiss Imager.Z1 AX10 microscope (Oberkochen, Germany) with CytoVision v.7.1 software. Images were analyzed and quantified using ImageJ v.1.53 software (https://imagej.net/, downloaded on 1 October 2022). Regarding the evaluation of the co-localization of TLR9 and LAMP1, ImageJ was used with the JACoP plug-in.

### 2.10. Lentivirus Transduction

MDA-MB-231 cells were plated in 6-well plates (1 × 10^5^ cells/well) in 1.5 mL of DMEM medium with 500 µL of green fluorescent protein (GFP)–lentivirus (Lv-GFP) suspension with 6 µg/mL polybrene (Hexadimethrine bromide, H9268, Sigma-Aldrich, St. Louis, MA, USA) for 24 h. After the incubation, the culture medium was refreshed, and selection was performed with 1 µg/mL puromycin (A11138-03, Gibco, Waltham, MA, USA) for 96 h. The percentage of MDA-MB-231-GFP-labeled cells was confirmed by flow cytometry using a BD Accuri^TM^ C6 flow cytometer (Becton Dickinson). MDA-MB-231-GFP cells were subsequently cultured for 10 days (short-term) and 4 weeks (long-term) in control conditions and with 10 ng/mL of Early-cfDNA-Gluc, Early-cfDNA-NoGluc, Late-cfDNA-Gluc, and Late-cfDNA-NoGluc.

### 2.11. Co-Culture Growth Advantage Assay

MDA-MB-231-GFP cells selected with Early-cfDNA-Gluc, Early-cfDNA-NoGluc, Late-cfDNA-Gluc, or Late-cfDNA-NoGluc were 1:1 co-cultured with parental unlabeled MDA-MB-231. Cells were exposed for 48 h to the respective cfDNA variant. Flow cytometry was used to measure the proportion of MDA-MB-231-GFP versus parental MDA-MB-231. Data were acquired in a BD Accuri^TM^ C6 flow cytometer from Becton Dickinson and processed with BD Accuri Software v1.0.34.1.

### 2.12. Statistical Analysis

GraphPad Prism 7 software was used to conduct the statistical analyses (https://www.graphpad.com/, downloaded on 1 September 2022). Three biological replicates were used for each treatment (N = 3). The sample data were reported as mean (normal distribution) ± SD. A two-tailed unpaired Student’s *t*-test was used to compare data from each group. Multiple comparisons were performed using one-way ANOVA or two-way ANOVA with Dunnett’s or Tukey’s test, with *p* < 0.05 being statistically significant. MetaboAnalyst 5.0 (assessed on 1 August 2024) was used for multivariate statistical analysis of ^1^H-NMR data, with metabolite concentrations as inputs and Pareto-scaling. MetaboAnalyst 5.0 was used to generate heatmaps reflecting the univariate analysis of the extracellular levels of the various metabolites discovered by NMR, with the Euclidean distance measure and the Ward cluster algorithm serving as the analysis parameters.

## 3. Results

### 3.1. The Consumption of Glucose by MDA-MB-231 Is Significantly Affected by cfDNA Exposure

**Figure 1:**
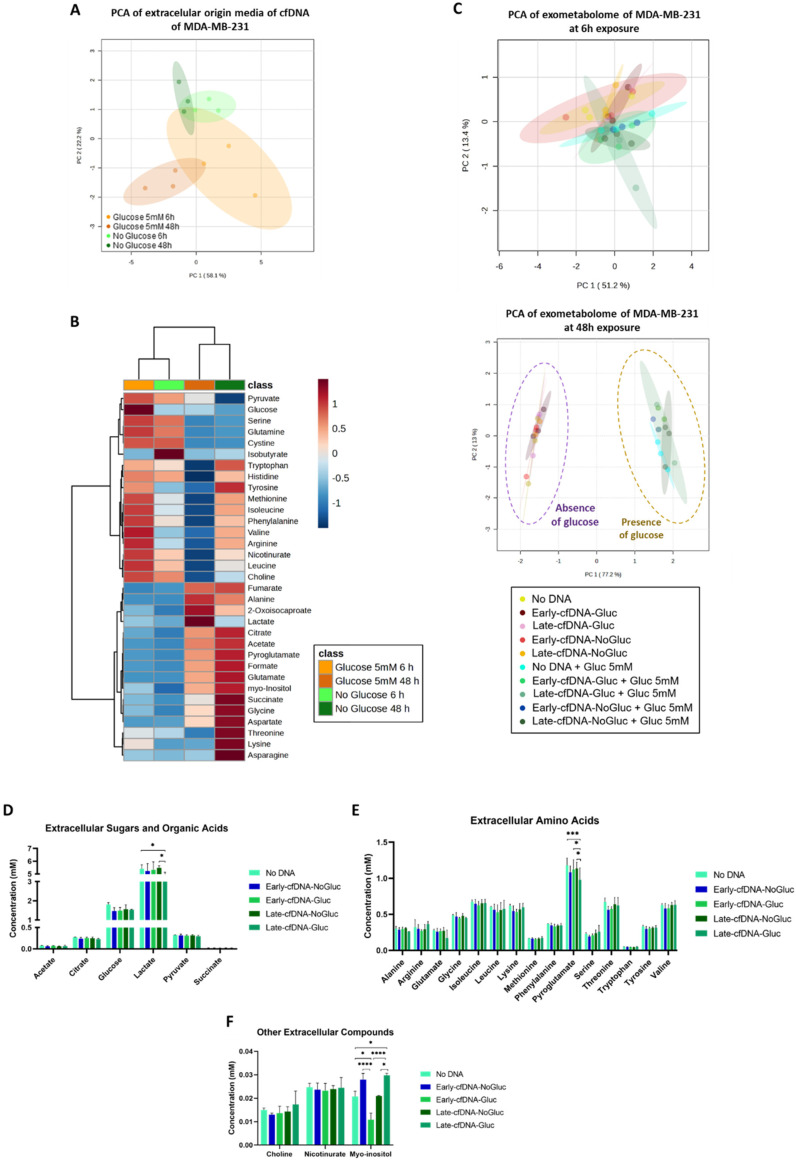
The consumption of glucose by MDA-MB-231 is significantly affected by cfDNA exposure. Cells were cultured for 6 and 48 h in the presence or absence of glucose (5 mM). (**A**) The Principal Component Analysis (PCA) score plot presents the metabolic profile clustering pattern of the media from which the cfDNA variants were isolated concerning the bioavailability of glucose and the time of incubation. Four variants were isolated: Early-cfDNA-Gluc, Early-cfDNA-NoGluc, Late-cfDNA-Gluc, and Late-cfDNA-NoGluc. (**B**) Heatmap showing the concentrations of the identified metabolites on MDA-MB-231 supernatants. New MDA-MB-231 cells were exposed to 10 ng/mL of each variety of cfDNA for 48 h in the presence of glucose (5 mM). Exometabolome in conditioned media was defined by nuclear magnetic resonance (^1^H-NMR). (**C**) The Principal Component Analysis (PCA) score plot presents the metabolic profile clustering pattern of cells exposed to Early-cfDNA variants and Late-cfDNA variants. The detected compounds were organized in groups. (**D**) Extracellular concentration of detected sugars and organic acids. (**E**) Levels of extracellular amino acids. (**F**) Other metabolites detected. Results are represented as mean ± SD. Two-way ANOVA was applied, followed by Tukey’s test, considering * *p* < 0.05, *** *p* < 0.001, and **** *p* < 0.0001.

The exometabolome of the cells of origin of cfDNA variants (Early-cfDNA-Gluc, Early-cfDNA-NoGluc, Late-cfDNA-Gluc, or Late-cfDNA-NoGluc) was defined to assess if glucose availability and cell culture duration (6 h and 48 h) would impact MDA-MB-231 cells’ metabolic profile. Through ^1^H-NMR, it was verified that both variables affect the exometabolome of the MDA-MB-231 cells. The Principal Component Analysis (PCA) showed that glucose availability and scarcity were separated by the second component, while cultures of 6 h tended to separate from the 48 h cultures by the first component (Figure 1A). Upon 48 h in glucose availably condition, the decrease in glucose levels was accompanied by an extracellular lactate accumulation (Figure 1B). After 48 h, glutamine was seen to be depleted from the media, showing that it was taken up by cells both in the presence and absence of glucose (Figure 1B). Leucine, isoleucine, and valine, which are branched-chained amino acids (BCAAs), were less concentrated in conditioned media from cell cultures exposed to glucose at 48 h but not yet at 6 h (Figure 1B). Moreover, cells exposed to glucose for 48 h showed a decrease in extracellular arginine levels (Figure 1B). Heatmap representation of the univariate analysis performed on the extracellular concentrations (Figure 1B) indicates that 6 h cultures have more similarities between them (Glucose 5 mM and No Glucose) than to their respective 48 h counterparts.

New MDA-MB-231 cells were exposed to the cfDNA variants in the presence and absence of glucose, and 28 metabolites were identified in the conditioned culture media. With 6 h of cfDNA exposure, no evident separation was seen between the conditions tested, suggesting that the exometabolome at 6 h was not yet significantly affected. However, the metabolic profiles of cells treated with cfDNA for 48 h clustered according to the absence and presence of glucose, independently of the cfDNA type (Figure 1C). This suggests that glucose as a carbon source influences the exometabolome of MDA-MB-231 cells. Since differences were observed at 48 h, we further studied the impact of the different cfDNAs on exometabolome in the presence of glucose. Regarding glucose levels, the exometabolome of all cells exposed to cfDNA variants tended to present lower levels of glucose compared with No DNA control cells (Figure 1D). The Late-cfDNA-Gluc variant induced a significant decrease in lactate compared with No DNA control and with Late-cfDNA-NoGluc (Figure 1D). Pyroglutamate levels tended to decrease in cells exposed to cfDNA variants, being significantly decreased upon exposure to Late-cfDNA-Gluc compared with all the other culture conditions (Figure 1E). The exometabolome of cells exposed to Late-cfDNA-Gluc also tended to have lower levels of alanine and glutamate compared with the other culture conditions (Figure 1E). The levels of myo-inositol were significantly altered in cells exposed to all cfDNA variants except for Late-cfDNA-NoGluc. This way, Early-cfDNA-Gluc significantly decreased myo-inositol levels, while Early-cfDNA-NoGluc and Late-cfDNA-Gluc significantly increased myo-inositol levels (Figure 1F).

### 3.2. Late-cfDNA-NoGluc and Late-cfDNA-Gluc Tended to Decrease MDA-MB-231 Cell Death Levels and Increase Migration in the Presence of Glucose

**Figure 2:**
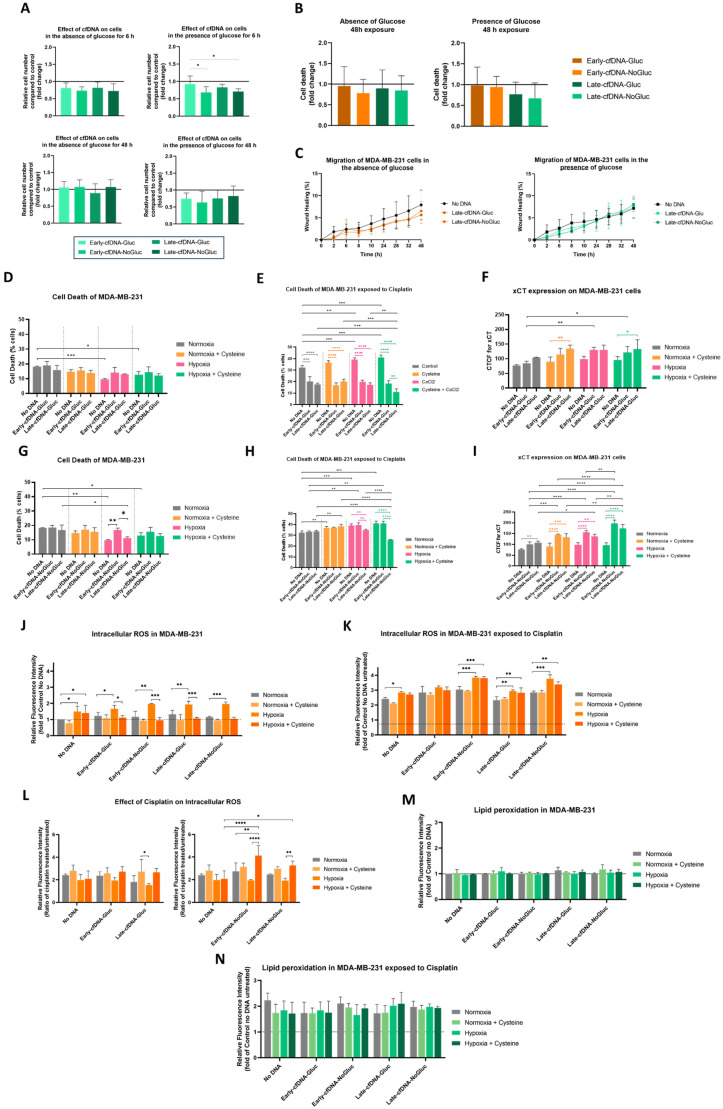
Early-cfDNA-Gluc and Late-cfDNA-Gluc promote cisplatin resistance and protect MDA-MB-231 cells from oxidative stress. Cells were cultured with or without glucose and subjected to treatment with the different cfDNA variants (10 ng/mL). (**A**) The relative number of MDA-MB-231 cells exposed to Early-cfDNA-Gluc, Early-cfDNA-NoGluc, Late-cfDNA-Gluc, or Late-cfDNA-NoGluc in the absence and presence of glucose (fold change to No DNA control). (**B**) Cell death evaluated by flow cytometry in MDA-MB-231 exposed to Early-cfDNA-Gluc, Early-cfDNA-NoGluc, Late-cfDNA-Gluc, or Late-cfDNA-NoGluc for 48 h (fold change to No DNA control). (**C**) Wound healing assay using MDA-MB-231 cells exposed to Late-cfDNA-Gluc or Late-cfDNA-NoGluc and in No DNA control in the absence or presence of glucose. (**D**,**E**) Cell death was evaluated by flow cytometry in high glucose (25 mM) conditions following treatment in normoxic and hypoxic (10 mM CoCl_2_) conditions with or without 4.2 mM cysteine. Cell death was evaluated in cells exposed to Early-cfDNA-Gluc and Late-cfDNA-Gluc with no drugs (**D**) and with (25 µg/mL) cisplatin exposure (**E**). xCT expression in MDA-MB-231 cells exposed to (**F**) Early-cfDNA-Gluc and Late-cfDNA-Gluc under normoxic and hypoxic environments with and without cysteine. Cell death was evaluated in cells exposed to Early-cfDNA-NoGluc and Late-cfDNA-NoGluc with no drugs (**G**) and with (25 µg/mL) cisplatin exposure (**H**). (**I**) xCT expression in MDA-MB-231 cells exposed to Early-cfDNA-NoGluc and Late-cfDNA-NoGluc under normoxic and hypoxic environments with and without cysteine. Flow cytometry analysis of intracellular ROS levels in MDA-MB-231 cells exposed to Early-cfDNA-Gluc, Early-cfDNA-NoGluc, Late-cfDNA-Gluc, or Late-cfDNA-NoGluc in normoxic and hypoxic, with and without cysteine, in the no drugs or cisplatin conditions. Cells exposed to cfDNA variants with no drug (**J**) and with (25 µg/mL) cisplatin exposure (**K**). (**L**) Ratio of ROS between cells, cisplatin-treated and untreated, in all culture conditions. Flow cytometry analysis of lipid peroxide levels in the presence of glucose in MDA-MB-231 cells exposed to Early-cfDNA-Gluc, Early-cfDNA-NoGluc, Late-cfDNA-Gluc, or Late-cfDNA-NoGluc, cultured in normoxic and hypoxic with and without cysteine, upon no drugs (**M**) and cisplatin (**N**) conditions. Results are represented as mean ± SD. One and two-way ANOVA were applied, followed by Tukey’s test, considering * *p* < 0.05, ** *p* < 0.001, *** *p* < 0.001, and **** *p* < 0.0001.

The effect of cfDNA on cancer cell proliferation was assessed at 6 and 48 h of cell culture in MDA-MB-231 cells with No cfDNA and exposed to the cfDNA variants in the absence and presence of glucose. In the absence of glucose, no significant differences were observed either at 6 h or at 48 h (Figure 2A). In the presence of glucose, there seems to be a general tendency to decrease cell proliferation with cfDNA exposure, with significant differences in cells exposed to Early-cfDNA-Gluc and Late-cfDNA-NoGluc at 6 h when compared with the No DNA control (Figure 2A).

Since no significant cell proliferation results were obtained at 48 h, we proceeded to evaluate the cell death of MDA-MB-231 at this timepoint. In the absence of glucose, no significant effect was verified upon exposure to cfDNA variants when compared with the No DNA control condition (Figure 2B). In the presence of glucose, cells exposed to Early-cfDNA-NoGluc and Early-cfDNA-Gluc showed no differences in cell death levels, but cells exposed to Late-cfDNA-NoGluc and Late-cfDNA-Gluc tended to decrease cell death levels (Figure 2B).

Afterward, Late-cfDNA-NoGluc and Late-cfDNA-Gluc were evaluated in regard to their effect on MDA-MB-231 migration in the absence and presence of glucose. In the absence of glucose, both Late-cfDNA-NoGluc and Late-cfDNA-Gluc tended to decrease migration (Figure 2C). Nevertheless, the cells maintained in glucose availability inverted the tendency and increased migration over time (Figure 2C).

### 3.3. Early-cfDNA-Gluc and Late-cfDNA-Gluc Promoted Cisplatin Resistance in MDA-MB-231 Cells, Which Is Favored by Hypoxia and Cysteine Availability

Our previous studies proved the role of cysteine in protecting cells from hypoxia and prompting chemoresistance in cancer [28,29,30]. Therefore, we investigated if cfDNA has a role in chemoresistance and if it is affected by hypoxia (mimicked with CoCl_2_) and/or cysteine bioavailability. MDA-MB-231 cells were cultured in normoxia, normoxia + cysteine, hypoxia, or hypoxia + cysteine.

Cells exposed to Early-cfDNA-Gluc and Late-cfDNA-Gluc showed no differences in cell death within each culture condition (Figure 2D). Nevertheless, cells cultured in hypoxia with or without cysteine tended to present lower levels of cell death compared with normoxia and normoxia with cysteine, with these differences being significant in No DNA control cells (Figure 2D).

In No DNA control MDA-MB-231 cells, cisplatin treatment increased cell death, which was enhanced by hypoxia and by the presence of cysteine in normoxia and hypoxia (Figure 2E). Importantly, MDA-MB-231 cells exposed to Early-cfDNA-Gluc and Late-cfDNA-Gluc presented significantly lower levels of cell death compared with No DNA control in all culture conditions (Figure 2E). Moreover, in hypoxia, the protective effect of Late-cfDNA-Gluc was significantly improved by the presence of cysteine (Figure 2E). In cells cultured in hypoxia with cysteine, Late-cfDNA-Gluc was significantly more efficient than Early-cfDNA-Gluc in reducing cell death (Figure 2E).

Regarding cell death levels in the absence of cisplatin and upon exposure to Early-cfDNA-NoGluc and Late-cfDNA-NoGluc, it was observed that Early-cfDNA-NoGluc significantly increased the levels of cell death in hypoxia compared with No DNA control cells (Figure 2G). In the presence of cisplatin, it was observed that Late-cfDNA-NoGluc significantly decreased cell death in hypoxia, and this protection was enhanced by cysteine compared with the No DNA control cell in these culture conditions (Figure 2H).

### 3.4. Cysteine Enhances the Protection of MDA-MB-231 Cells from Oxidative Damage by Early-cfDNA-Gluc and Late-cfDNA-Gluc

Chemoresistance has been correlated to the expression of the cyst(e)ine antiporter xCT by Lewerenz et al. [31], and we observed that Late-cfDNA-Gluc and Late-cfDNA-NoGluc protection against cisplatin was improved by cysteine in hypoxia. Therefore, we evaluated the expression levels of xCT by immunofluorescence in cells exposed to cfDNA-Gluc (Early-cfDNA-Gluc and Late-cfDNA-Gluc) and cfDNA-NoGluc (Early-cfDNA-NoGluc and Late-cfDNA-NoGluc) variants, the same culture conditions. It was observed that xCT levels are significantly increased in MDA-MB-231 cells exposed to all cfDNA variants in all culture conditions, except Early-cfDNA-Gluc in normoxia compared to No DNA control (Figure 2F,I).

Cisplatin presents two mechanisms of action: the formation of DNA and protein adducts and the generation of reactive oxygen species (ROS) [32]. Thus, we then explored if cfDNA variants affected the intracellular ROS levels in normoxia and hypoxia with and without cysteine. Firstly, in cells exposed to no drug, hypoxia increased ROS levels in cells cultured with No DNA and with all cfDNA variants (Figure 2J). Interestingly, in hypoxia with cysteine, No DNA control cells still had increased levels of ROS, but cells exposed to all cfDNA variants rescued the levels of ROS compared to hypoxia without cysteine (Figure 2J). Upon cisplatin treatment, intracellular ROS in MDA-MB-231 increased in hypoxia with and without cysteine for No DNA control cells and for cells exposed to all cfDNA variants, except in Early-cfDNA-Gluc (Figure 2K). Considering the cisplatin effect and the contribution of each cfDNA and condition to the ROS levels (Figure 2L), it was clear to see that only the cfDNA-Gluc variants indicate cysteine protection since Hypoxia + Cysteine conditions decrease ROS ratio between cisplatin-treated and untreated cells (Figure 2L). Accordingly, cfDNA-NoGluc variants presented an increased ROS level ratio with hypoxia in the presence of cysteine (Figure 2L).

Cisplatin-induced ROS can react with membrane lipids, inducing lipid peroxidation, which can lead to ferroptosis [33,34]. Hence, we investigated the levels of lipid peroxidation, and cells exposed to cisplatin presented increased levels of lipid peroxides (Figure 2N) compared with cells without drugs (Figure 2M), regardless of cfDNA exposure. However, no differences were observed in the levels of lipid peroxides in any culture conditions regarding cfDNA exposure, normoxia and hypoxia, or cysteine availability (Figure 2M,N).

### 3.5. The Early-cfDNA-NoGluc Variant Induced a Metabolic Remodeling to Support Chemoresistance

**Figure 3:**
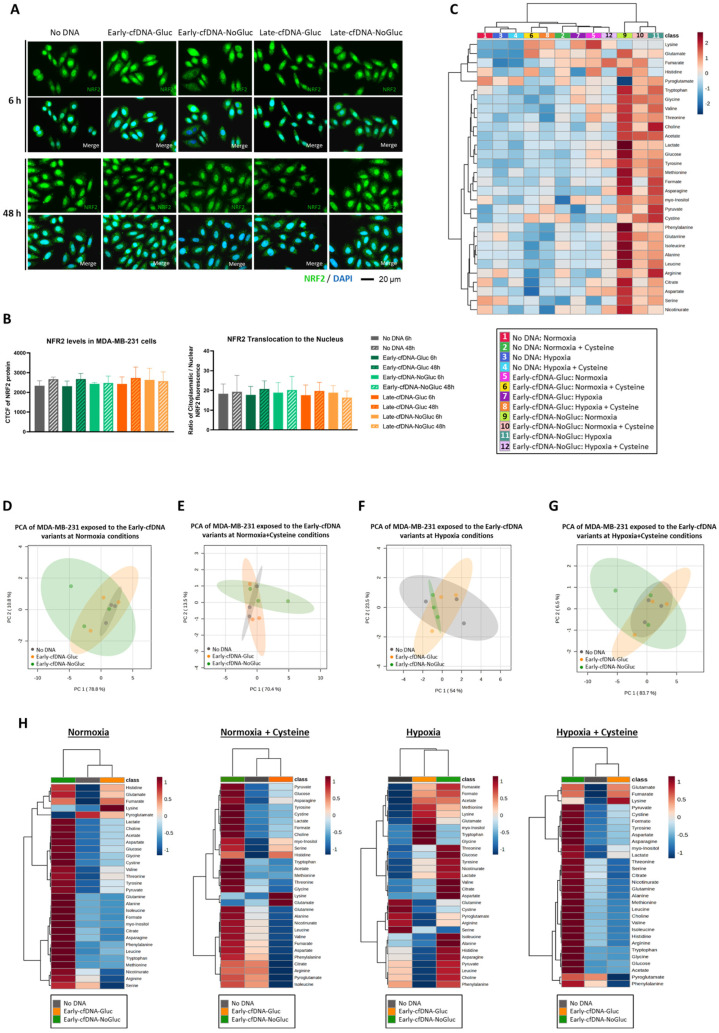
Metabolic adaptation is essential for chemoresistance, as verified with the Early-cfDNA-NoGluc variant. Cells were cultured with or without glucose and subjected to treatment with the different cfDNA variants (10 ng/mL). (**A**) Immunofluorescence for detection of NRF2 (green); the nuclei are dyed with DAPI (blue). (**B**) Quantification of the NRF2 immunofluorescence, represented by the ratio of cytoplasmatic protein over nucleic protein. (**C**) Heatmap showing the concentrations of the identified metabolites on MDA-MB-231 supernatants when cells were treated with cisplatin, exposed to the cfDNA variants and conditions of normoxia and hypoxia, with and without cysteine supplementation. (**D**) Principal Component Analysis (PCA) of normoxia conditions. (**E**) Principal Component Analysis (PCA) of normoxia with cysteine supplementation conditions. (**F**) Principal Component Analysis (PCA) of hypoxia conditions. (**G**) Principal Component Analysis (PCA) of hypoxia with cysteine supplementation conditions. (**H**) Heatmaps with the concentrations of the metabolites in the supernatants separated by normoxia, normoxia with cysteine supplementation, hypoxia, and hypoxia cysteine supplementation. Results are represented as mean ± SD. One-way ANOVA was applied, followed by Tukey’s test.

NRF2 has been described as a cellular protector from oxidative stress [35,36]; thus, NRF2 protein levels were evaluated upon exposure to cfDNA (Figure 3A). Interestingly, when evaluating the NRF2 translocation to the nucleus, no differences were verified when the cells were exposed to the different cfDNA variants (Figure 3B).

Additionally, considering the results obtained in the cell death assessment with cisplatin (Figure 2E,H), we further evaluated the metabolic remodeling associated with the tested conditions. All cfDNA variants reduced cisplatin-induced cell death in hypoxic conditions with cysteine supplementation, except for Early-cfDNA-NoGluc. Since Early-cfDNA-NoGluc was associated with cisplatin susceptibility, ^1^H-NMR analysis of the extracellular media was performed in cells exposed to Early-cfDNA-NoGluc, along with cells exposed to Early-cfDNA-Gluc and No DNA cells. A total of 30 metabolites were detected and quantified. Globally, Early-cfDNA-NoGluc conditions showed higher amino acid concentrations compared to the other conditions (Figure 3C). The impact of the cfDNAs in normoxia or hypoxia, with or without cysteine supplementation, was analyzed. The PCA did not indicate clear separations between the cfDNA variants independently of the environmental setting (Figure 3D–G). Nevertheless, when evaluating the extracellular concentration of the metabolites in detail, we saw that in normoxia, normoxia with cysteine supplementation, and hypoxia with cysteine supplementation, the Early-cfDNA-NoGlu confers a distinct exometabolome once it clusters apart from the other conditions in the heatmaps (Figure 3H).

### 3.6. Long-Term Selection with Late-cfDNA-NoGluc and Late-cfDNA-Gluc Variants Induced Quiescence in MDA-MB-231 Cells

**Figure 4:**
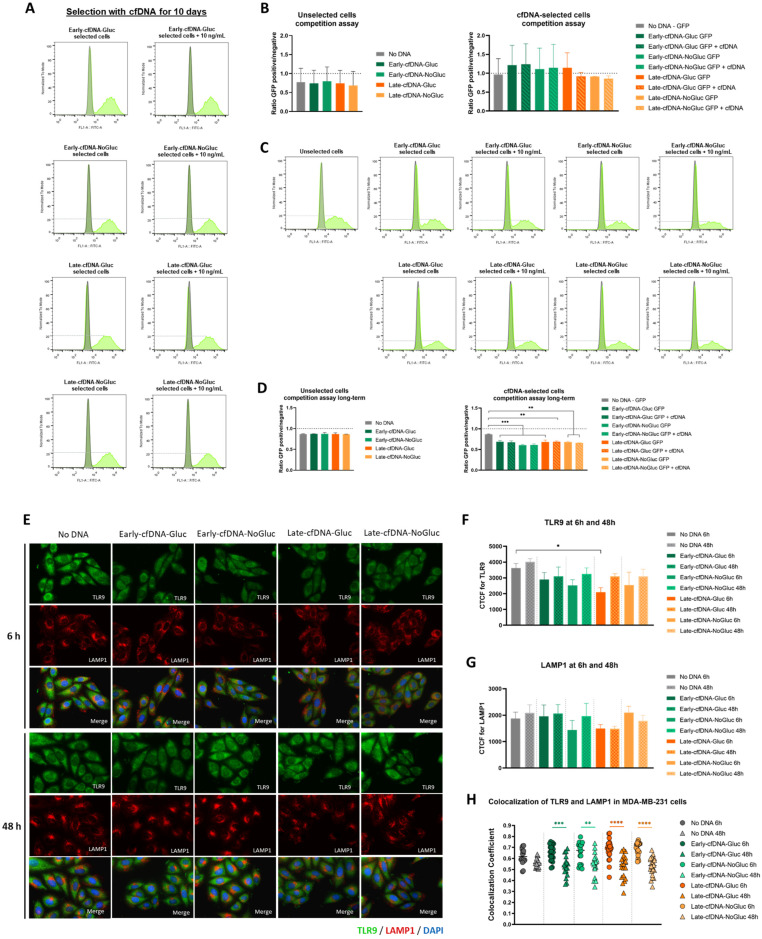
Late-cfDNA variants promote a decrease in the proliferative behavior in MDA-MB-231 cells upon long-term selection. (**A**) Population plots analyzed by flow cytometry for the short-term selection with MDA-MB-231 unselected cells in grey and MDA-MB-231-GFP cells in green. (**B**) Competition assays of unselected cells upon cfDNA variant (10 ng/mL) stimulation (on the right) and in selected cells for 10 days with and without cfDNA variant (10 ng/mL) exposure (on the left). Results are represented as the ratio of the GFP-positive cells over the GFP-negative cells. (**C**) Population plots analyzed by flow cytometry for the long-term selection with MDA-MB-231 unselected cells in grey and MDA-MB-231-GFP cells in green. (**D**) Competition assays of unselected cells upon cfDNA variant (10 ng/mL) stimulation (on the right) and in selected cells for 4 weeks with and without cfDNA variant exposure (on the left). Results are represented as the ratio of the GFP-positive cells over the GFP-negative cells. (**E**) Immunofluorescence for detection of TLR9 (green) and LAMP1 (red); the nuclei are dyed with DAPI (blue). (**F**) Quantification of the Corrected Total Cell Fluorescence (CTCF) of TLR9 in cells exposed to the cfDNA variants for 6 or 48 h. (**G**) Quantification of the CTCF of LAMP1 in cells exposed to the cfDNA variants (10 ng/mL) for 6 or 48 h. (**H**) Pearson’s correlation coefficient for the colocalization analysis between TLR9 and LAMP1 proteins. Results are shown as mean ± SD. * *p* < 0.05, ** *p* < 0.001, *** *p* < 0.001, and **** *p* < 0.0001. One-way ANOVA was used, followed by Tukey’s test.

To further understand the impact of cfDNA variants in proliferation, unselected MDA-MB-231 cells were co-cultured with GFP-MDA-MB-231 cells that were previously selected with cfDNA. After 10 days of cfDNA exposure, it was observed that no variant seemed to significantly impact the proliferation capacity of the cells since the GFP positive/negative ratio was maintained (Figure 4A,B). When evaluating a longer exposure period to the cfDNA variants (with continuous stimulus), we verified a significant decrease in the GFP positive/negative ratio in the MDA-MB-231 co-cultures (Figure 4C,D).

The expression of the TLR9 and LAMP1 was assessed in MDA-MB-231 cells exposed to the cfDNA variants to evaluate if TLR9 activation led to its translocation to endolysosomes (Figure 4E). Interestingly, cfDNA exposure showed a tendency to decrease TLR9 levels, with the decrease being significant in cells exposed to Late-cfDNA-Gluc for 6 h (Figure 4F). Regarding LAMP1 levels, no significant differences were seen with the cfDNA exposure, although a decreased tendency could be observed in cells exposed to Late-cfDNA-NoGluc and Late-cfDNA-Gluc variants (Figure 4G). At 6 h, the co-localization of TLR9 and LAMP1 did not differ between control cells and cells exposed to cfDNA (Figure 4H). Interestingly, at 48 h, cells exposed to all the cfDNA variants presented a decrease in the co-localization of TL9 and LAMP1 compared with the control (Figure 4H).

## 4. Discussion

In recent years, the clinical relevance of cfDNA in oncology has sparked substantial interest. Several studies have established the potential of cfDNA as a biomarker for cancer diagnosis and monitoring [11,37,38]. Still, the importance of cfDNA in cancer biology and progression remains unknown. The cfDNA present in cancer patients’ peripheral blood serum has been shown to activate signaling pathways [39], such as through toll-like receptors (TLRs), for example [40].

The cfDNA could have a role in the adaptive capacity of cancer cells to harsh environments. These difficulties arise throughout cancer’s course and trigger the selection of the malignant cells capable of continuing their expansion. However, the tumor masses are known to be heterogeneous, and their heterogeneity impairs the ability to adapt to the tumor microenvironment. This effect causes the sensitivity and resistance to vary between cancer cell subsets within the same tumor. As a result, the cfDNA is a tool that could facilitate communication among cancer cells in order to orchestrate adaptive processes.

The effect of cfDNA generated from MDA-MB-231 cells, a TNBC cell line, on metabolic remodeling and cisplatin resistance was investigated in this study, taking into account variations in glucose bioavailability and culture length of time. We categorized the cell culture in the absence of glucose as an unfavorable metabolic environment and the cell culture in the presence of glucose as an ideal and advantageous metabolic environment. The cfDNA release could represent the result of cell distress or an attempt to sustain survival.

Because glucose is considered a crucial fuel for tumor growth, we studied the alterations in the metabolic reprogramming triggered by glucose bioavailability. MDA-MB-231 cells showed different metabolic profiles both at 6 h and at 48 h after being in the presence and absence of glucose (Figure 1A). Lactate secretion in response to glucose intake suggests glucose catabolism (Figure 1B), implying that the glycolysis process was active. Glutamine was depleted from the media after 48 h, showing that cells consumed it both in the presence and absence of glucose (Figure 1B). Glutamine has been demonstrated to be the most rapidly utilized amino acid by tumor cells [41]. It is a crucial amino acid for sustaining ATP synthesis, redox balance, and signal transduction activities [42] and is intracellularly metabolized to glutamate, which is then transformed to α-ketoglutarate via the tricarboxylic acid (TCA) cycle. Furthermore, glutamine-derived glutamate is the main source of amino acid synthesis, mainly through transamination processes [43,44]. As the tumor grows, different amino acids are needed to sustain cancer cell abilities, also serving as possible substitute fuel sources for cells to maximize nutrient use [45]. BCAAs were consumed more by cells exposed to glucose at 48 h but not yet at 6 h (Figure 1B). This observation fits the fact that BCAAs play a vital role in the proliferation of cancer cells by serving as nitrogen donors that are essential for the synthesis of nucleotides [46,47], in addition to serving as alternate sources that can power the TCA cycle [48]. The decrease in arginine levels in cell cultures with glucose at 48 h (Figure 1B) suggested a role for arginine in cancer metabolic adaptation. Arginine serves as a precursor for polyamines [49,50], which play a pivotal role in tumor progression by enhancing DNA synthesis and promoting cell proliferation [50]. Additionally, polyamides provide a protective effect on nucleic acids, safeguarding them from damage and ensuring DNA stability [51,52]. L-arginine serves as a precursor to nitric oxide (NO) [53], a crucial signaling molecule that governs various cellular processes, influences tumor growth, facilitates extracellular matrix remodeling, and plays a significant role in angiogenesis [54,55].

When MDA-MB-231 cells were exposed to cfDNA, it was verified that at 6 h, metabolic differences were not yet seen, but at 48 h, the cells cultured with cfDNA and glucose presented a distinct metabolic profile from the ones with glucose scarcity (Figure 1C). This effect was independent of the cfDNA variant, but the exposure to cfDNA stimulated glucose consumption (Figure 1D). The same trend was seen for pyroglutamate (Figure 1 E), indicating that glutamine-dependent metabolism is also enhanced by cfDNA. The Late-cfDNA-Gluc variant reduced lactate, alanine, and glutamate levels (Figure 1D,E), the pivotal metabolites of glucose and glutamine-dependent pathways. Lactate results from glycolysis, and it is a valuable energetic source that can be imported by cancer cells to supply oxidative phosphorylation (OXPHOS). On the other hand, lactate also supports glucose synthesis [56]. Alanine is a gluconeogenic amino acid [57], which works as a source of pyruvate to supply the synthesis of glucose [56] and OXPHOS. Glutamate, as mentioned above, derives mainly from glutamine degradation and sustains TCA cycle function and other amino acid synthesis [43,44]. Overall, these results reinforce the role of cfDNA as a signaling molecule, whose relevance is observed in the metabolic remodeling needed for cancer cells to thrive and face stressful conditions in the tumor microenvironment.

Because metabolic adaptation supports all the biological processes, it was interesting to notice that all the cfDNA variants induced distinct effects on cancer cell features. The enhancement of tumor heterogeneity is closely linked to cell proliferation [58], as better-adapted cells proliferate more quickly and outcompete maladapted cells. In the absence of glucose, cfDNA did not affect the proliferation rate of MDA-MB-231 cells, but in the presence of glucose for 6 h, Early-cfDNA-NoGluc and Late-cfDNA-NoGluc variants retarded the proliferation rate. Importantly, at 48 h of cell culture, MDA-MB-231 cells exposed to all cfDNA variants presented similar proliferative rates (Figure 2A). Therefore, cells cultured in glucose scarcity (selective disadvantage) release cfDNA that can aid other cancer cells to adapt their metabolism and rescue the proliferative capacity in order to maintain tumor growth, which is why at 48 h, MDA-MB-231 cells exposed to Early-cfDNA-NoGluc and Late-cfDNA-NoGluc reached a similar proliferation rate to MDA-MB-231 cells exposed to cfDNA isolated from cells cultured with glucose. The observed distinct effects at 6 h and 48 h indicate that this process involves a process of adaptation upon cfDNA stimulation.

Surgery, radiation therapy, and/or chemotherapy are the standard treatments for BC. TNBCs are particularly susceptible to DNA-damaging agents like platinum salts, as these cancers often exhibit a high frequency of defects in DNA repair mechanisms [59]. However, acquired chemoresistance presents a major challenge in oncology, as changes in key regulatory pathways, such as PI3K/AKT/mTOR, contribute to tumor resistance to conventional therapies [60]. Our previous work in other cancer models highlighted the critical role of cysteine in hypoxia adaptation, which underlies chemoresistance [28,29,30]. Cysteine’s importance in cancer cell survival is attributed to its function as a carbon and sulfur source and as a precursor to the antioxidant glutathione (GSH), with elevated GSH levels being associated with chemotherapy resistance [61,62]. Notably, in the absence of cisplatin, cysteine supplementation consistently reduced ROS levels, while hypoxia significantly elevated them. As previously mentioned, cysteine is a precursor of GSH [63,64], and hypoxic conditions are known to induce oxidative stress [65,66]. Interestingly, under hypoxic conditions, cysteine was able to reduce ROS levels when cfDNA was present, further supporting the role of cfDNA in metabolic remodeling and cell survival under stress. Cisplatin treatment significantly increased ROS levels across all tested conditions (Figure 2K,L). However, in hypoxia, cysteine could not reduce ROS accumulation during cisplatin treatment, possibly due to the elevated ROS burden. Comparing cells exposed to cisplatin with Early-cfDNA-NoGluc and Late-cfDNA-NoGluc in hypoxia—both with and without cysteine supplementation—revealed that Late-cfDNA-NoGluc was more effective at reducing ROS levels. Additionally, all cfDNA variants reduced cisplatin-induced cell death under hypoxic conditions with cysteine supplementation, except for Early-cfDNA-NoGluc. This suggests that cells cultured without glucose for an extended period were more capable of releasing cfDNA that modulates cancer cell metabolism and survival. This is consistent with the proliferation assay results (Figure 2A).

NRF2, a key transcription factor regulating the antioxidant response, controls cysteine uptake and the expression of GSH production enzymes [67]. Our results demonstrated that both Early-cfDNA-Gluc and Late-cfDNA-Gluc variants protect cells from cisplatin-induced toxicity, as evidenced by lower levels of cell death compared to other culture conditions. Notably, during oxidative stress induced by platinum salts, oxidized cfDNA can enter cancer cells, stimulating antioxidant mechanisms and enhancing NRF2 expression [68,69,70,71]. Except for Early-cfDNA-NoGluc, all cfDNA variants conferred cisplatin resistance, although no significant differences in NRF2 expression or its translocation to the nucleus were observed (Figure 3A,B). Similarly, no differences were found in xCT expression (Figure 2F,I), an acyst(e)ine transporter whose expression is known to be modulated by NRF2 and has been linked to chemoresistance [31]. The lack of significant variation in xCT expression between cfDNA variants suggests that differential chemoresistance modulation is likely not dependent on xCT regulation. It is noteworthy, however, that the susceptibility-related variant (Early-cfDNA-NoGluc) generally exhibited higher xCT levels (Figure 2I). Furthermore, although xCT has been a focal point in cancer metabolism studies, cystine can also be transported into cells via alternative transporters, such as solute carrier family 3 member 1 (rBAT, *SLC3 A1*), which does not require glutamate export [71].

The exometabolome analysis confirmed differences in the metabolic profiles of cisplatin-treated MDA-MB-231 not exposed to cfDNA, Early-cfDNA-NoGluc, and Early-cfDNA-Gluc, both cultured in normoxia and hypoxia. These findings are particularly noteworthy since Early-cfDNA-NoGluc was compared to Early-cfDNA-Gluc, which originated from cells with the same culture duration but under different glucose availability conditions. As observed, Early-cfDNA-Gluc and Early-cfDNA-NoGluc present a distinct exometabolome (Figure 3H), with cells exposed to Early-cfDNA-Gluc presenting a quite constant metabolic profile in normoxia and normoxia with cysteine (Figure 3H), which is disturbed upon hypoxia for a further rescue upon hypoxia with cysteine. Thus, Early-cfDNA-Gluc controls the metabolic balance efficiently. Regarding Early-cfDNA-NoGluc, the exometabolomes of MDA-MB-231 cells exposed to this cfDNA variant are variable depending on the normoxia and hypoxia condition with or without cysteine supplementation (Figure 3H), indicating that metabolic remodeling induced by cfDNA requires adaptation. Therefore, the metabolic profiles suggest that Early-cfDNA-NoGluc influences metabolic adaptation in a context-dependent manner. Higher extracellular amino acid levels indicate that cells exposed to Early-cfDNA-NoGluc may restrict γ-glutamyl cycle activity or use alternate amino acid transport pathways. The γ-glutamyl cycle has been shown to sustain amino acid availability in hypoxic conditions [72]. Our data suggest that cfDNA exposure may disrupt this mechanism, resulting in selective amino acid retention in the extracellular environment rather than increased cellular absorption. Consequently, the pro-tumoral power of released cfDNA depends on the environmental context, selective pressure, and metabolic drift.

Regarding the ability of cfDNA to induce selective advantage to MDA-MB-231 cells, results indicate that a short selection time was not enough to take any effect; however, increasing the duration of continuous exposure to cfDNA resulted in a lower ratio of selected over unselected cells. Such implies a lower proliferative rate of selected cells and suggests that all cfDNA variations may induce a quiescent state in MDA-MB-231 cells (Figure 4A–D). DNA-induced growth inhibition in yeasts has already been documented [73,74], but there are still no reports of cfDNA playing a pro-quiescent role in cancer cell settings. Quiescent cells are thought to be the drivers of cancer metastasis and recurrence, and they are more resistant to anti-cancer treatments [75,76]. Although they are thought to be in a reduced number within the tumor, dormant cancer stem cells can revive disease in a more aggressive form [77,78,79]. The quiescence induction is in agreement with the tendency to increase the migration of cells exposed to Late-cfDNA-NoGluc and Late-cfDNA-Gluc in cells cultured in glucose scarcity (Figure 2C). Cell division and migration cannot occur at the same time within the same cell, and their coordination must be tightly regulated [80,81,82]. The migration–proliferation dichotomy has been explored in cancer, and it seems to be a characteristic accounting for disease success, stating that when cells proliferate, their migratory capacity is suspended, being afterward reactivated when cell division is stopped [83]. Moreover, the combined effect of cell proliferation rate and sugar metabolism has been reported to be a critical condition explaining the so-called SICD (sugar-induced cell death) in yeast [84]. This is an important subject deserving further investigation in cancer cells.

The activation of signaling is needed for the modulatory role of cfDNA released from certain cancer cells to act on other cancer cells in the same tumor. In this context, TLR9 is an interesting candidate in mediating cfDNA-induced phenotypic remodeling. With all cfDNA variants exposure, there was a trend to an overall decrease in TLR9 levels, which can be due to the activation of TLR9 cascade and consequent proteolysis [85,86]. TLR9 is mostly located in intracellular vesicles within the endoplasmic reticulum, and after being stimulated by ligands such as CpG dinucleotides, TLR9 translocates through the Golgi complex to late LAMP-1^+^ endolysosomes [87,88]. Still, after the exposure to cfDNA, the co-localization index between TLR9 and LAMP1-late endosomes dropped from 6 h to 48 h. These results may suggest the instant activation of TLR9 at 6 h, describing cfDNA uptake and processing as a rapid and perhaps fleeting process due to the receptor proteolysis.

**Figure 5:**
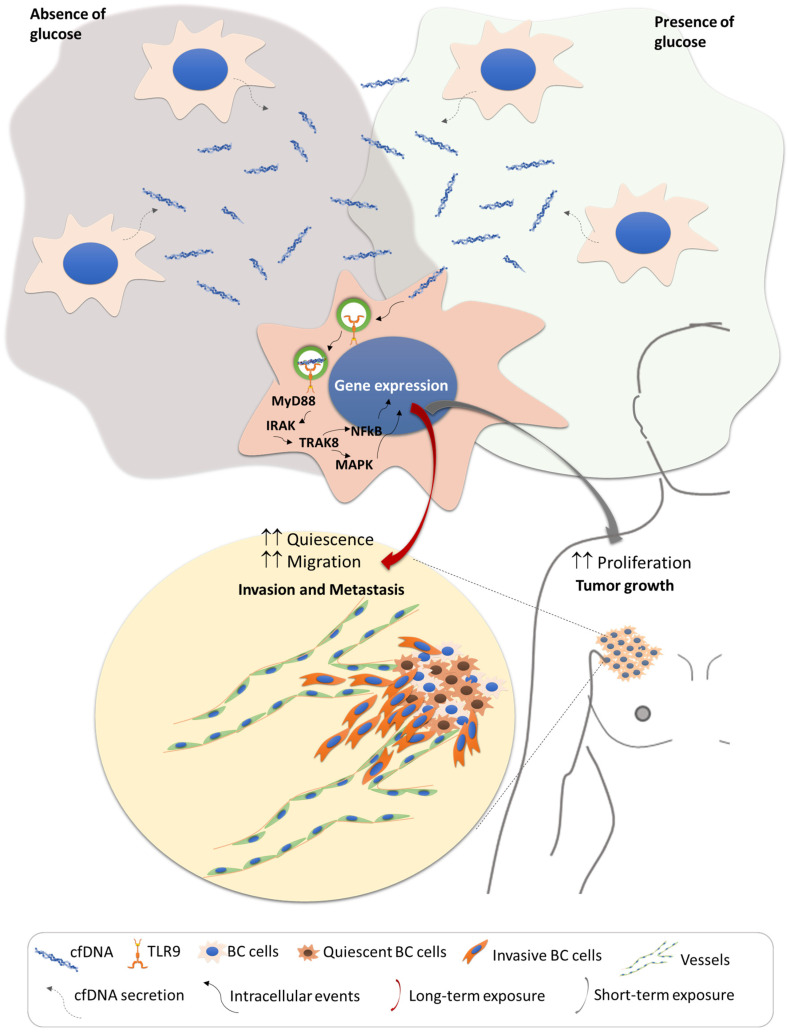
The complex and multifaceted role of cfDNA in cancer biology. The exposure to cfDNA induces a metabolic remodeling that influences the proliferative capacity of breast cancer (BC) cells, accounting for primary tumor growth. A long-term selection with cfDNA prompts the quiescent state of BC cells concomitantly with increased migratory capacity, and both phenomena are associated with cancer metastasis, chemoresistance, and recurrence. Signaling activated by cfDNA probably occurs through TLR9. The stress or beneficial conditions in the tumor microenvironment will influence the pro-tumoral power of cfDNA for the control of cancer progression, depending on the context accounting for increased (↑↑) quiescence, migration and proliferation.

Overall, these results underscore the complex and multifaceted role of cfDNA in cancer biology (Figure 5). The ability of cfDNA to modulate metabolic reprogramming, cell proliferation, and quiescence could significantly impact cancer progression and resistance to treatment. Furthermore, the activation of signaling pathways, potentially through TLR9, suggests a mechanistic basis for the phenotypic changes observed in cfDNA-exposed cells. Taken together, these findings highlight cfDNA as a key player in the tumor microenvironment, facilitating communication between cancer cells and supporting their survival and adaptation under stress, thereby contributing to tumor progression and therapy resistance.

## Data Availability

Data is available in the public repository https://github.com/lgafeira/SelfDNA_MDA (accessed on 25 January 2025).
